# Expressed sequence tag analysis of guinea pig (*Cavia porcellus*) eye tissues for NEIBank

**Published:** 2008-12-19

**Authors:** Mukoma F. Simpanya, Graeme Wistow, James Gao, Larry L. David, Frank J. Giblin, Kenneth P. Mitton

**Affiliations:** 1 Eye Research Institute, Oakland University, Rochester, MI; 2 Section on Molecular Structure and Functional Genomics, National Eye Institute, National Institutes of Health, Bethesda, MD; 3 Department of Biochemistry and Molecular Biology of the School of Medicine, Oregon Health Sciences University, Portland, OR

## Abstract

**Purpose:**

To characterize gene expression patterns in guinea pig ocular tissues and identify orthologs of human genes from NEIBank expressed sequence tags.

**Methods:**

RNA was extracted from dissected eye tissues of 2.5-month-old guinea pigs to make three unamplified and unnormalized cDNA libraries in the pCMVSport-6 vector for the lens, retina, and eye minus lens and retina. Over 4,000 clones were sequenced from each library and were analyzed using GRIST for clustering and gene identification. Lens crystallin EST data were validated using two-dimensional electrophoresis (2-DE), matrix assisted laser desorption (MALDI), and electrospray ionization mass spectrometry (ESIMS).

**Results:**

Combined data from the three libraries generated a total of 6,694 distinctive gene clusters, with each library having between 1,000 and 3,000 clusters. Approximately 60% of the total gene clusters were novel cDNA sequences and had significant homologies to other mammalian sequences in GenBank. Complete cDNA sequences were obtained for many guinea pig lens proteins, including αA/αAinsert-, γN-, and γS-crystallins, lengsin and GRIFIN. The ratio of αA- to αB-crystallin on 2-DE gels was 8: 1 in the lens nucleus and 6.5: 1 in the cortex. Analysis of ESTs, genome sequence, and proteins (by MALDI), did not reveal any evidence for the presence of γD-, γE-, and γF-crystallin in the guinea pig. Predicted masses of many guinea pig lens crystallins were confirmed by ESIMS analysis. For the retina, orthologs of human phototransduction genes were found, such as *Rhodopsin*, *S-antigen* (*Sag*, *Arrestin*), and *Transducin.* The guinea-pig ortholog of *NRL*, a key rod photoreceptor-specific transcription factor, was also represented in EST data. In the ‘rest-of-eye’ library, the most abundant transcripts included decorin and keratin 12, representative of the cornea.

**Conclusions:**

Genomic analysis of guinea pig eye tissues provides sequence-verified clones for future studies. Guinea pig orthologs of many human eye specific genes were identified. Guinea pig gene structures were similar to their human and rodent gene counterparts. Surprisingly, no orthologs of γD-, γE-, and γF-crystallin were found in EST, proteomic, or the current guinea pig genome data.

## Introduction

The study of eye disease depends upon experimental animals to elucidate disease mechanisms, as well as to find preventative and/or therapeutic options. The guinea pig has long been a valuable animal model for studying various tissues of the eye including cornea, lens, and retina, as well as various eye disorders. For example, vision researchers have taken advantage of the fact that guinea pigs, like humans, require vitamin C in their diet and thus can be made scorbutic. Human eye tissues such as aqueous humor, lens, and cornea contain high levels of ascorbate, up to 10 mM [1], and the guinea pig is an ideal animal model to investigate the ocular antioxidant role of this vitamin. Thus, guinea pigs have been used to study the role of vitamin C in protecting against sugar-induced cataract [2,3], inhibiting UVB-induced effects on the cornea and lens [4–6], and healing injuries to the cornea caused by heat [7].

It has also been suggested that the guinea pig is possibly the best non-primate model for investigating certain aspects of human cataractogenesis [8]. Unlike mice and rats, lenses of the guinea pig display certain key biochemical similarities to the human lens. For example, guinea pig lenses do not undergo significant oxidation of protein sulfhydryl residues as they age [9–11]. Furthermore, the guinea pig lens contains high concentrations of a UVA chromophore, not kynurenine as in the human, but NADPH bound to ζ-crystallin [12,13]. Thus, it has been possible to use guinea pigs as a model for exploring the possible role of UVA light in contributing to human maturity-onset nuclear cataract [14,15]. Guinea pigs are also similar to humans in that they develop increased lens nuclear light scattering and myopia after lengthy treatment with hyperbaric oxygen [16–18]. The guinea pig has been used to investigate various effects of in vivo hyperoxia on lens crystallins, cytoskeletal proteins, membrane proteins and lipids, as well as on levels of antioxidants such as glutathione, cysteine, and ascorbate [17,19–21]. The O_2_-induced effects were similar to those occurring to proteins, lipids, and antioxidants in the aging human lens. Age-related truncation of MIP/aquaporin 0 also occurs in the guinea pig lens, particularly in the nucleus as it does in the human, and this truncation is accelerated by in vivo O_2_-treatment [19,22].

Guinea pigs are also frequently used for research on the retina. Unlike several other experimental animals (rats, mice, cats, dogs, and rabbits), the guinea pig is born with eyes open, and can be studied by electroretinogram (ERG) analysis at birth [23]. Since key aspects of fetal retinal development in the guinea pig and human are very similar, this species is a useful model to study the effects of adverse intrauterine conditions on retinal development [24,25]. Guinea pig retinas have been employed to show that Müller cells in the vertebrate retina act like optical fibers, funneling light through the inner retina to light-detecting photoreceptors [26]. The avascular nature of the guinea pig retina makes it a useful model for studying retinal oxygen consumption [27] and for investigating changes in vitreous pO_2_ levels following enzymatically-induced posterior vitreous detachment [28]. Also, guinea pigs have been used to evaluate the protective effect of vitamin E against retinal edema during ischemia-reperfusion injury [29].

Guinea pigs have been employed for research on the cornea, iris, and trabecular meshwork. Kannan et al. [30] used guinea pigs to evaluate the effects of galactose feeding and aging on the uptake of glutathione by the cornea. Both guinea pig and human corneal epithelium contain significant activities of xanthine oxidoreductase and xanthine oxidase, which under certain pathological states may contribute to oxidative corneal damage [31,32]. Guinea pigs have also been useful for investigating nerve terminal impulses generated by cold sensitive receptors in the cornea [33], and for evaluating the protective effects of various agents against corneal infection [34]. Guinea pigs are similar to humans in that both adrenergic and cholinergic innervations are present in the iris dilator muscle [35,36]. Also, unlike many mammals such as the rabbit, the guinea pig possesses an ocular structure very similar to human trabecular meshwork, and is useful for studies of neurotransmitters that may participate in aqueous humor regulation and control of intraocular pressure [37,38].

Although the guinea pig is an important model species for eye research, and is currently the subject of a genome sequencing project, there is a general lack of expressed sequence tag (EST) analysis of any guinea pig tissues. At the time of this writing, there is no gene structural and transcript annotation for the guinea pig genome, and EST data are required to confirm gene structures (introns, exons) and post-transcriptional splicing. Only one other eye-derived cDNA library (whole eye) has been published [39], and this did not concentrate on the lens or retina.

We made cDNA libraries from three guinea pig eye tissues, including the lens, retina, and ‘rest-of-eye’ (eye minus lens and retina), as part of the NEIBank project. Over 4,000 clones from each library were subjected to single pass sequencing and the resulting ESTs grouped into clusters for gene identification. Nearly 60% of the total gene clusters had significant homologies to mammalian sequences (non-guinea pig) in GenBank. Also, as a tissue particularly rich in crystallin proteins, guinea pig lenses were extracted and analyzed by electrospray ionization mass spectrometry (ESIMS) to validate EST data from the library protocol. Surprisingly, EST data suggested an absence of several γ-crystallins. The cDNA-predicted masses of crystallin proteins were in good agreement with the actual protein masses as determined by ESIMS. Each library contained enough ESTs to demonstrate alternative splicing events for several key tissue-specific genes. The guinea pig NEIBank cDNA libraries provide valuable data for studying gene expression, structure and splicing, for vision scientists and the guinea pig genome project.

## Methods

### Animal care

All animal care and other work performed in this study conformed to the US Department of Agriculture standards and the ARVO statement for the use of animals in ophthalmic and vision research. Hartley guinea pigs were obtained from the Kuiper Rabbit Ranch (Indianapolis, IN) and Elm Hill laboratories (Chelmsford, MA). Euthanization of the animals was conducted using CO_2_ asphyxiation.

### Isolation of guinea pig eye tissue mRNA

Twelve eyes from six 2.5-month-old guinea pigs were removed and divided into anterior and posterior portions by cutting along the ora serrata. The anterior segment containing the cornea, lens, iris, ciliary body, and trabecular meshwork was lifted away from the posterior eyecup. The lens was then separated from the anterior segment. Neural retina was carefully removed from the eyecup, leaving the retinal pigment epithelium (RPE), choroid, sclera, and optic nerve behind. Harvested tissues were transferred immediately into 5 volumes of RNA*later* solution (Ambion, Austin, TX) at 4 °C and frozen at −70 °C until RNA extraction. Three different tissue groups were used for RNA isolation: (i) lens, (ii) retina, and (iii) eye minus lens and retina (this tissue included cornea, iris, ciliary body, trabecular meshwork, choroid, sclera and RPE). The libraries created were designated lens (clone code letters: nbb), retina (naz), and eye minus lens and retina (nba).

### Guinea pig eye tissue cDNA library construction

Total RNA was extracted from the three guinea pig eye tissues with RNAzol (Tel-Test Inc., Friendswood, TX). mRNA was prepared by oligo(dT) cellulose affinity chromatography and cDNA was synthesized and cloned into SalI-Not I sites of the pCMVSport-6 vector (Invitrogen, Carlsbad, CA) as previously described [40]. Libraries were not normalized or amplified.

### Sequence and data analysis

For each cDNA library, plasmid DNAs were prepared from several thousand individual clones and processed for single pass sequencing in the NIH Intramural Sequencing Center. High quality cDNA sequences were analyzed using BLAST (Basic Local Alignment Search Tool) program [41] (National Center for Biotechnology Information [NCBI], National Library of Medicine, Bethesda, MD) to compare with GenBank nucleotide sequences, protein sequences (non-redundant) and the database of expressed sequence tags (ESTs) [42]. A custom software package, GRIST (GRouping and Identification of Sequence Tags) [42], was used to group ESTs into “gene clusters” of overlapping cDNA sequence, and to identify each cluster based upon BLAST results. To confirm some identities, and to compare splicing patterns between guinea pig and human, some groups were analyzed by BLAT analysis (with the human genome) [43] and visualization in EyeBrowse, an eye-centric version of the UCSC Genome Browser [44,45]. Information on all the sequenced guinea pig eye tissue clones and clusters is deposited at the NEIBank website.

### Guinea pig ocular morphology

Eyes from 20-month-old guinea pigs were fixed in PBS containing 4% paraformaldehyde and 20% isopropanol for 24 h and processed for paraffin sections. Whole globe cross sections were stained with hematoxylin/eosin reagent and photographed with a Nikon Optiphot-2 microscope equipped with a digital camera (SPOT; Diagnostic Instruments, Sterling Heights, MI).

### 2-DE gels of guinea pig lens cortical and nuclear proteins

Analysis of lens cortical and nuclear water soluble (WS) proteins from 2.5-month-old guinea pigs was conducted using two dimensional electrophoresis (2-DE). The lenses were frozen rapidly in crushed dry ice and separated into equatorial cortex (the periphery of the lens) and nucleus (the center of the lens) with the use of a 2.5 mm cork borer. The tissues were homogenized (100 mg wet weight of lens per ml buffer) at 4 °C in a N_2_ atmosphere in a 20 mM sodium phosphate buffer (pH 7.0) containing 1 mM EDTA. The homogenate was centrifuged for 25 min at 20,000x g to isolate WS proteins. Protein concentration was determined with a bicinchoninic assay (BCA) protein assay (Pierce Biotechnology, Rockford, IL), using BSA as the standard. 2-DE was conducted as previously described [46–48] by isoelectric focusing (IEF) lens WS proteins using self-poured immobilized pH gradient (IPG) gel strips (18 cm, pH 5–9 NL) produced using Immobiline II reagents (GE Healthcare, Piscataway, NJ). IPG strips were rehydrated overnight in 400 μl rehydration solution containing 400 µg soluble lens protein as previously described [47,48]. IEF was performed on an electrophoresis apparatus (Protean IEF cell; Bio-Rad Laboratories, Hercules, CA), the second dimension separation performed on 23x20 cm, 12% SDS–PAGE gels, proteins stained with Coomassie G250, and gels images scanned as described elsewhere [49]. Image analysis of gels was then performed using computer software, Image J. To determine the relative abundance of α-crystallins, the spots were delineated, integrated grayscale intensities determined, and the background of each spot subtracted by performing a similar analysis in a nearby region containing no protein. For mass spectrometry analysis, protein spots from 2-DE gels were excised, trypsinized, and analyzed by matrix assisted laser desorption (MALDI) to acquire 10 MS/MS spectra from each digest as previously described [49]. Sequest (ThermoFinnigan, San Jose, CA) searches to match MS/MS data to peptide sequences were performed using a guinea pig database containing 1,138 entries, including the sequences of guinea pig crystallins generated in this study, and appended with sequence reversed entries to assess the false discovery rate. MS/MS results were filtered so that Xcorr and ΔCN values were greater than 1.5 and 0.05, respectively, and two peptides matched to a single protein entry. Using these criteria, there were no matches to the sequence reversed entries.

### Analysis of intact guinea pig lens cortical crystallin masses by ESIMS

Lenses were harvested from 2.5-month-old guinea pigs, frozen immediately in crushed dry ice and divided into cortex and nucleus as described above. The isolated lens “cylinder” (containing the nucleus plus anterior and posterior cortex) was discarded, and the remaining equatorial cortex (70% of the total lens weight) was homogenized, centrifuged to isolate WS protein, and protein concentration determined as described above.

Lens cortical WS proteins were separated using 2-DE as described above, except that pH 3–10 nonlinear immobilized pH gradient gels strips were used (GE Healthcare, Piscataway, NJ), and second dimension SDS–PAGE gels were negatively stained with imidazole-zinc [50]. A total of 29 stained protein spots were excised from duplicate gels. Mass measurement of proteins eluted from 2-DE gels was performed as previously described [46], with the following modifications. Spots pooled from duplicate gels were shaken twice for 15 min in 192 mM glycine, 25 mM Tris base, 50 mM DTT, 0.1% SDS, and crushed by passing through a 20 µm stainless steel frit using a 0.5 ml gas tight syringe. One-hundred and fifty µl of 96 mM glycine, 12.5 mM Tris base, 50 mM DTT was then added to the syringe to transfer the remaining gel particles into a centrifuge tube, and the resulting slurry was shaken for 30 min. The slurry was then transferred to an Ultrafree-MC microcentrifuge filter (UFC30HV00, Millipore, Bedford, MA), centrifuged for 15 min at 13,000x g, an additional 50 µl of the above solution added, and the device centrifuged again. The collected liquid was then dried by vacuum centrifugation, redissolved in 50 µl of 5% formic acid, and the masses of the eluted proteins determined by injecting the sample onto a 1.0x250 mm C4 column. The same trap cartridge, column, and electrospray ionization technique was used as before [46], except that a 20 µl/min flow rate and 2%–60% acetonitrile gradient over 50 min was used, and 0.05% TFA was added to the mobile phase to prevent formation of SDS-protein adducts during mass analysis. A total of 15 of the 29 isolated spots were found to have sufficient amounts of protein for LC-MS analysis. Whole mass deconvolution was performed with BioWorks software (version 3.2; ThermoFisher, Waltham, MA), and measured masses compared to theoretical masses using Protein Analysis Work Sheet software (PAWS version 8.1.1, 1997; ProteoMetrics, LLC, New York, NY).

## Results and Discussion

### Guinea pig ocular tissues

**Figure 1:**
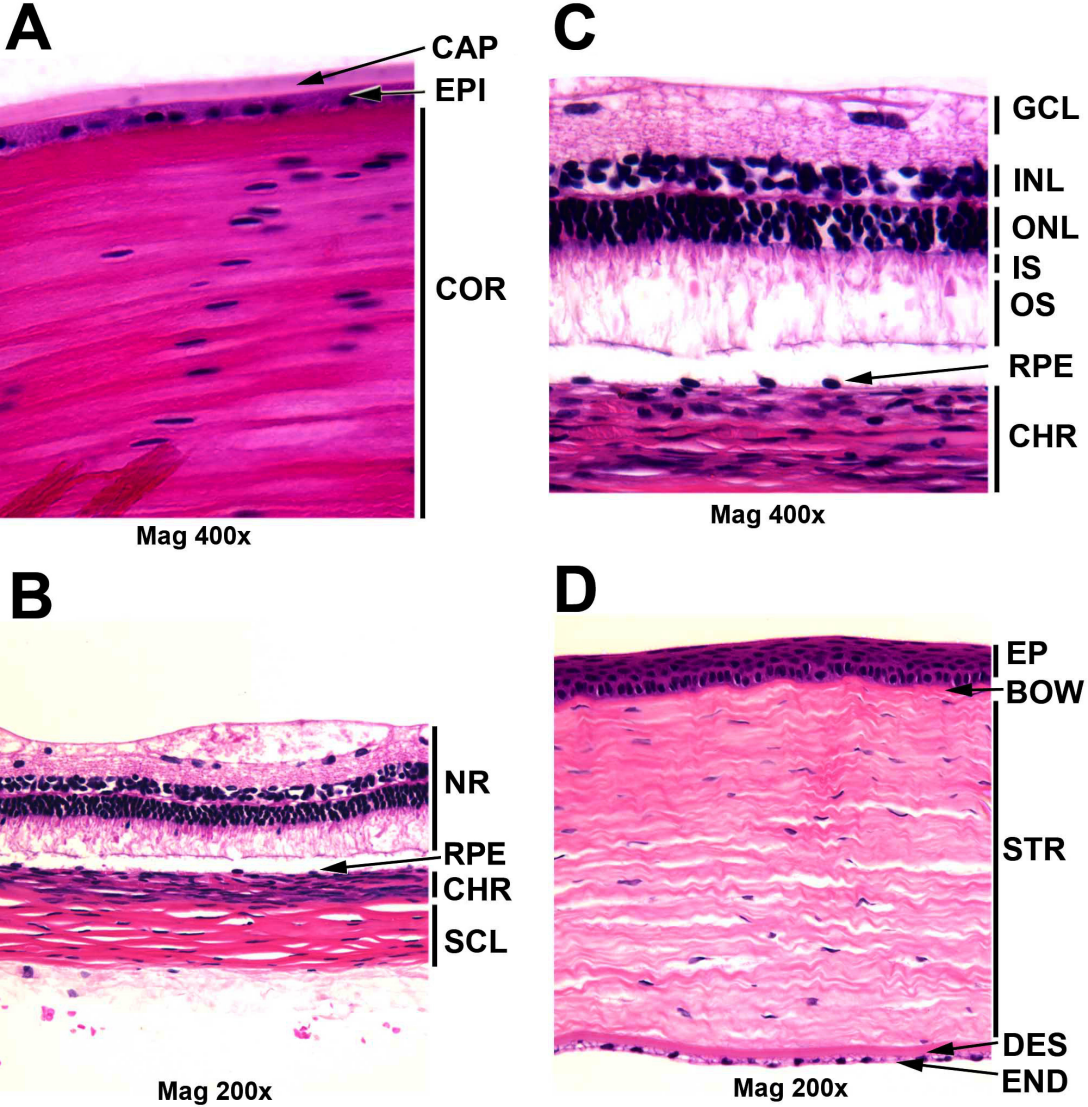
Morphology of guinea pig eye tissues for the Hartley strain used for the NEIBank library, stained with hematoxylin and eosin reagent. **A**: Lens capsule, epithelium and cortex in the bow region: capsule (CAP), epithelium (EPI), and cortical fiber cells (COR). The guinea pig lens is similar to human and mouse. **B**: Neural retina (NR), retinal pigment epithelium (RPE), choroid (CHR) and sclera (SCL). **C**: Retinal layer and choroid: ganglion cell layer (GCL), inner nuclear layer (INL), outer nuclear layer (ONL), inner segment (IS), outer segment (OS), retinal pigment epithelium (RPE), and choroid (CHR). The guinea pig retina is 4–5 nuclei thick, similar to the human retina. **D**: Cornea: corneal epithelium (EP), Bowman’s membrane (BOW), Stroma (STR), Descemet’s membrane (DES) and endothelium (END). The guinea pig cornea is similar to human, while the mouse has a thinner stroma.

Intact 2.5-month-old guinea pig lenses were used to make the lens cDNA library, designated “nbb.” The guinea pig lens is very similar to the human lens, with a monolayer epithelium, but the guinea pig capsule is thinner than the human capsule . Guinea pig lens capsule, epithelium, and cortex are shown in Figure 1A. Neural retina (Figure 1B,C) was used for the retina cDNA library, designated as “naz.” The guinea pig outer nuclear layer (ONL) is about 5 nuclei thick (photoreceptors), which is more similar to the human ONL (6 nuclei) than the mouse ONL (10–12 nuclei) [51,52]. The eye minus lens and retina cDNA library, NEIBank designation “nba,” included several eye tissues such as choroid (Figure 1B,C), sclera (Figure 1B), RPE (Figure 1B,C), iris, and cornea (Figure 1D).

### NEIBank guinea pig ESTs: novel cDNA and gene information

Novel genome assemblies, such as the current guinea pig genome project, require EST and mRNA sequence evidence to confirm gene structures, including intron and exon boundaries, and variable splicing of transcripts. Compared to human, mouse, and several other mammals, there is a paucity of EST data available for the guinea pig. Thus, the ESTs from the libraries described here are particularly valuable for ongoing annotation of the guinea pig genome, as well as for comparative genomics of mammalian eye tissues. ESTs were grouped into clusters (using GRIST). The percentages of guinea pig EST clusters having significant homologies to mammalian GenBank transcript sequences were 56% for retina, 69% for lens, and 55% for eye minus lens and retina. Most of the ESTs represent new information for the guinea pig transcriptome.

### Novel guinea pig lens cDNA sequences (nbb): absence of γD-F crystallins

As expected, crystallin genes accounted for a large fraction of the most abundant lens transcripts, with αA- and ζ-crystallin at very high levels (Table 1). Sequences for all the α- and β-crystallins (αA-, αAins-, αB-, βA1-, βA2-, βA3-, βA4-, βB1-, βB2-, βB3-crystallin) were observed, with reads giving complete or almost complete coverage of each transcript. Of the γ-crystallins, γS-crystallin was abundant and there were five clones for the recently identified γN-crystallin [53]. For the remainder, there were multiple clones for γA-, γB-, and γC-crystallin, but none for orthologs of γD-, γE-, and γF-crystallin (Table 1).

**Table 1:** Most abundant guinea pig lens cDNA transcripts (crystallins and non-crystallins [nbb]).

**Rank**	**GenBank description**	**N**
1	**alphaA-crystallin**	466
2	**zeta-crystallin**	330
3	**gammaS-crystallin**	183
4	**gammaB-crystallin**	113
5	**betaB2-crystallin**	113
6	**betaA4-crystallin**	112
7	**beta A3/A1-crystallin**	98
8	**betaB3-crystallin**	62
9	**gammaC-crystallin**	61
10	**betaB1-crystallin**	45
11	lengsin	27
12	phakinin (CP49, BFSP2)	23
13	**gammaA-crystallin**	22
14	carbonic anhydrase 3	22
15	major intrinsic protein (MIP)	20
16	GRIFIN	18
17	**alphaB-crystallin**	17
18	**betaA2-crystallin**	**1**4
19	filensin (BFSP1)	14
20	Glyceraldehyde 3-phosphate dehydrogenase (Gapdh)	13
21	ferritin light chain	12
22	elongation factor 1 alpha	12
23	ribosomal protein, large, P0	9
24	E-FABP (FABP5)	9
25	TPT1	9
26	cytochrome b5 reductase 1	9
27	alpha-enolase	8
28	tubulin, alpha 1	8
29	Serpin B6	8
30	ribosomal protein L4	7
31	prostaglandin-H2 D-isomerase	7
32	cyclin-G1	7
33	CD24 p	6
34	**gammaN-crystallin**	5
35	beta actin	5
36	laminin receptor	5
37	vimentin	5

Genes corresponding to the most abundant transcripts (≥5 clones) in 2.5-month-old guinea pig (*Cavia porcellus*) lens are listed and ranked by abundance. The total number of sequenced clones in each gene cluster (N) is indicated. Crystallins are shown in bold. GenBank descriptions are based on clusters having significant homologies to mammalian Genbank sequences.

**Figure 2:**
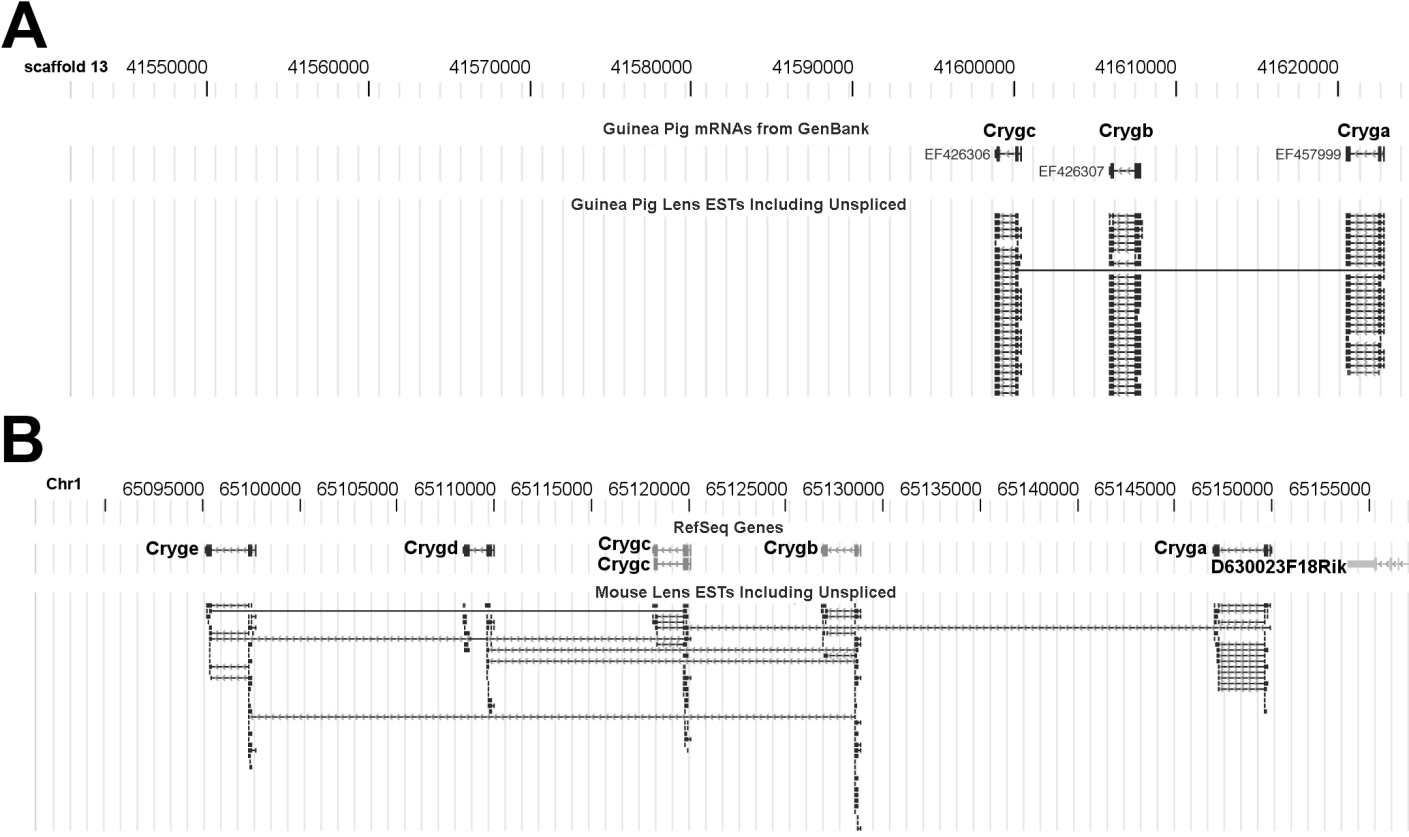
Absence of *Crygd, Cryde,* and *Crygf* in the guinea pig genome downstream of the *Cryga-*Crygc region. **A**: EyeBrowse view of NEIBank ESTs for guinea pig γ-crystallin genes, aligned to scaffold_13 of the guinea pig genome and mRNAs from GenBank. *Cryga* (EF457999), *Crygb* (EF426307) and *Crygc* (EF426306), were constructed from NEIBank EST data. There were no BLAT alignments to indicate the existence of guinea pig orthologs of the *Crygd, Cryde,* or *Crygf* genes. In this temporary scaffold, there were over 85,000 base pairs of gap-free DNA sequence downstream from *Crygc*. **B**: For comparison to the guinea pig, the mouse chromosome-1 region containing the γ-crystallin genes *Cryga* to *Cryge,* aligned to RefSeq (Reference Sequence) genes. Note: arrows indicate gene orientations, which are on the reverse strand.

**Figure 3:**
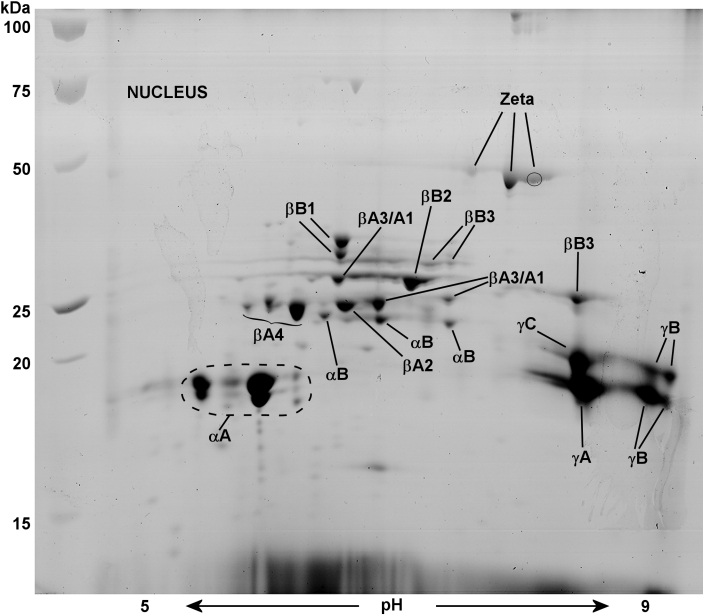
2-D Electrophoresis map showing identities of lens nuclear water-soluble proteins of a 2.5-month-old guinea pig. The major proteins of the lens nucleus were identified by matrix assisted laser desorption (MALDI) mass spectrometry. αA-crystallin was far more abundant than αB-crystallin (ratio of 8:1) as quantified by image analysis software, Image J. All β-crystallins (βA1-, βA2-, βA3-, βA4-, βB1-, βB2-, and βB3-crystallin) and γA-, γB-, and γC-crystallin were detected on 2-DE gels, but no protein signatures were found for γD-, γE-, or γF-crystallin. The gel contains 53 spots, 44 of which have been identified as various intact or truncated crystallins. Proteins were stained with Coomassie Blue G-250.

**Figure 4:**
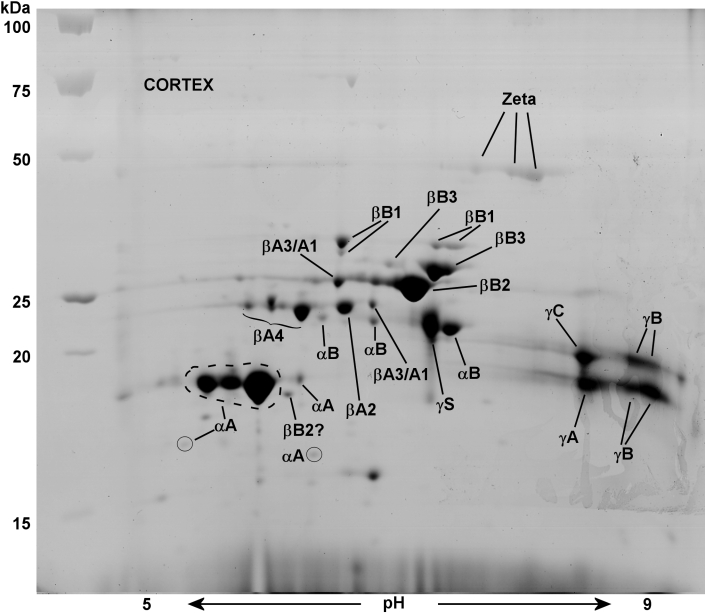
2-D Electrophoresis map showing identities of lens cortical water-soluble (WS) proteins of a 2.5-month-old guinea pig. The major proteins of the lens cortex were identified by MALDI mass spectrometry. The ratio of cortical αA-crystallin to αB-crystallin was 6.5:1 as quantified by image analysis software, Image J. All β-crystallins and γA-C-crystallin were detected, but no protein signatures were detected for γD-, γE-, and γF-crystallin. The gel contains 47 spots, 37 of which have been identified, with some proteins as intact or truncated crystallins. Proteins were stained with Coomassie Blue G-250. The symbol ? indicates presumptive protein identification.

ESTs for γA-, γB-, and γC-crystallins from guinea pig lens library are shown aligned with “scaffold_13” (Figure 2A) of the current guinea pig genome, and this is compared with a similar alignment in the mouse genome (Figure 2B). A scaffold is a portion of a genome sequence reconstructed from end-sequence whole genome shot gun clones. Scaffold_13 of the guinea pig genome is free of gaps for at least 85,000 base pairs downstream of γC-crystallin. Figure 2 illustrates the lack of EST or genomic sequences corresponding to γD- and γE-crystallin in guinea pig lens. In other mammals γF-crystallin *(Crygf)* is located further downstream from this region. In comparison, mouse γA- to γF-crystallin are fully contained within a region of only 56,000 base pairs. As described below, the absence of guinea pig ESTs for γD-, γE-, and γF-crystallin was supported by an absence of the corresponding guinea pig lens water-soluble proteins on 2-DE gels (Figure 3 and Figure 4). It is possible that the guinea pig has eliminated expression of γD-F-crystallin, perhaps by deletion of the genes themselves, or the genes are absent for this developmental stage of the guinea pig lens. Interestingly, searches of the current guinea pig genome have not yielded any gene sequences for these three crystallins (as of the October 2008, genome build). In humans, γE- and γF-crystallin are pseudogenes (present but not expressed) and γA- and γB-crystallin are expressed only at low levels.

**Figure 5:**
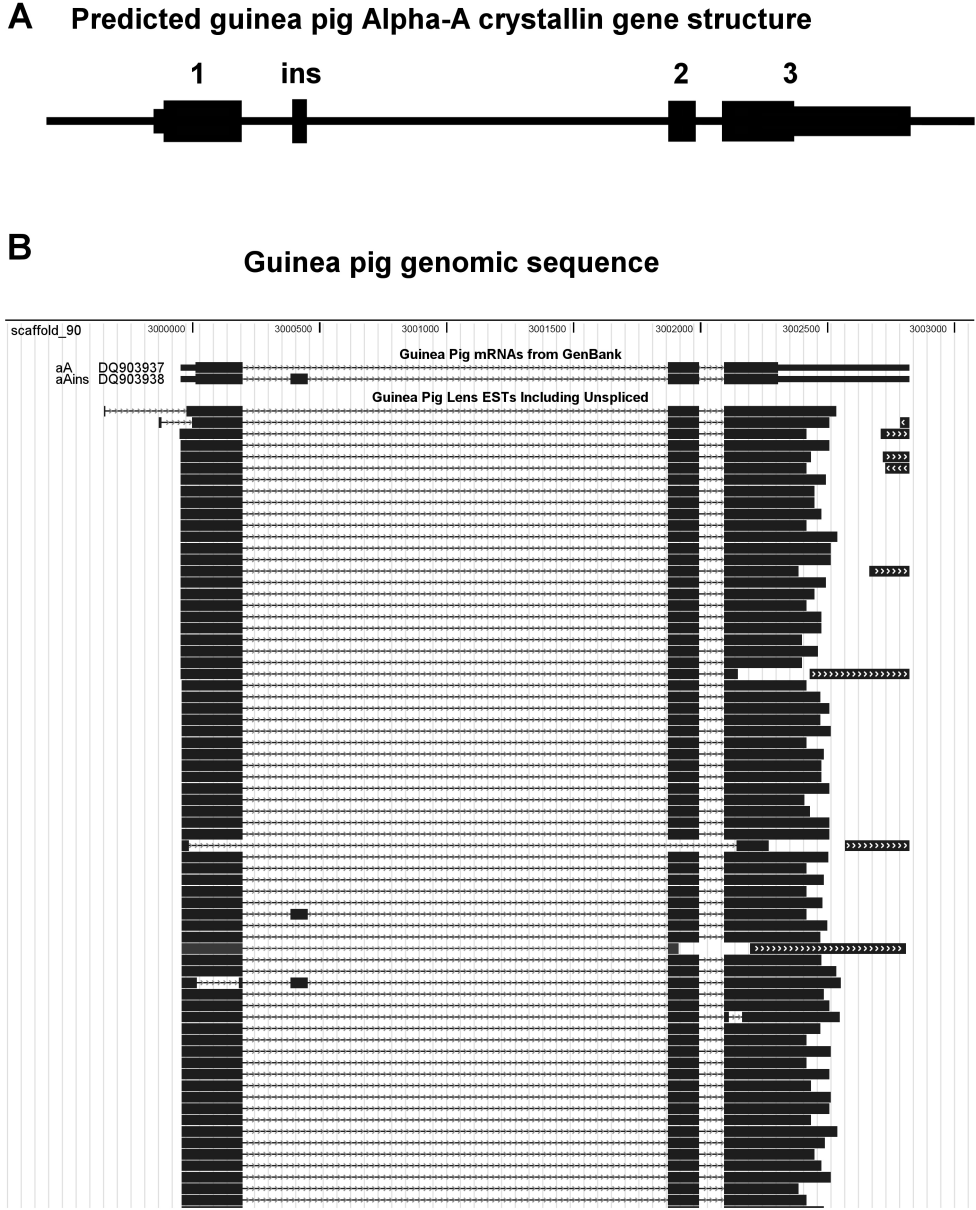
Alternate splicing of the guinea pig *α-crystallin* gene. **A**: Predicted guinea pig *α-crystallin* gene structure derived from EST data. **B**: Guinea pig lens *α-crystallin* ESTs including unspliced introns aligned to guinea pig genome (scaffold_90) and guinea pig mRNA from GenBank for *Cryaa* (DQ903937) and *Cryaa-ins* (DQ903938) viewed with EyeBrowse [48,49]. Note guinea pig “scaffolds” are not yet annotated for gene structure (build 7–17–08). Note: arrows show introns in the direction of sequence reads.

EST analyses with long high quality sequence reads can give complete coverage of abundant gene transcripts and can identify alternative transcripts. For instance, NEIBank lens ESTs were used to produce reference mRNA sequences for the guinea pig lens α-crystallins, *Cryaa* (DQ903937) and *Cryaa-ins* (DQ903938). Both are shown aligned to scaffold_90 of the guinea pig genome sequence (Figure 5). The gene structure of αA-crystallin and its minor component αAinsert-crystallin are identical except for an extra exon (23 amino acids) from alternative splicing of mRNA (Figure 5A,B). Full-length sequences for all the crystallin transcripts have been assembled and deposited in GenBank (Table 2).

**Table 2:** Full length open reading frame (orf) guinea pig mRNA sequences deposited in GenBank.

**Accession number**	**GenBank description**
DQ903937	alphaA-crystallin (Cryaa)
DQ903938	alphaAins-crystallin (Cryaa)
EU252027	alphaB-crystallin (Cryab)
EU306867	alpha-enolase (Eno1)
EU257502	alpha-transducin (Gnat1)
EF666995	betaA2-crystallin (Cryba2)
EF666994	betaA4-crystallin (Cryba4)
EF666993	betaB1-crystallin (Crybb1)
EF457998	betaB2-crystallin (Crybb2)
EF457997	betaB3-crystallin (Crybb3)
EU257501	beta-transducin (Gnb1)
EU327786	Carbonic anhydrase 3 (CA3)
EU868623	decorin (Dcn)
EU264005	E-FABP (FABP5)
EU827599	filensin (BFSP1)
EF457999	gammaA-crystallin (Cryga)
EF426307	gammaB-crystallin (Crygb)
EF426306	gammaC-crystallin (Crygc)
EU257500	gammaN-crystallin (Crygn)
DQ903939	gammaS-crystallin (Crygs)
EU862201	glyceraldehyde 3-phosphate dehydrogenase (Gapdh)
EU259197	GRIFIN
EU664998	keratin 12 (Krt12)
DQ324462	lengsin (Gludl1)
EU477759	leucine zipper transcription factor (NRL)
EU327787	major intrinsic protein (MIP)
EU827600	Mp19 (Lim2)
EU257503	phakinin (BFSP2
EF457995	rhodopsin (Rho)
EF457996	S-antigen (Sag)
EU330893	TPT1 (TPT1)
EU827601	vimentin (Vim)
EU833985	YB1 (YB1)

List of new and full length guinea pig (*Cavia porcellus*) open reading frame (ORF) mRNA sequences submitted to GenBank.

About 69% of the gene clusters had identities or homologies to sequences in GenBank (non-guinea pig). A gene cluster is a group of cDNA clones determined to be from the same gene based on overlapping sequences. Of the remaining ‘unidentified’ clones, the majority match positions in the (incomplete) guinea pig genome. Many of the ‘unidentified’ clones are probably from untranslated regions of guinea pig genes that do not have close matches in other species. Others may also have poor sequence quality that does not permit confident identification.

Similar to the guinea pig lens library, most abundant lens transcripts in the mouse lens library were αA-crystallin, β-crystallins (βA1-, βB1-, βB2-, and βB3-crystallin), and γ-crystallins (γB- and γS-crystallin) [54]. In contrast, the rat lens library had γ-crystallins (γA-F- and γN-crystallin) and some β-crystallins (βA1-, βA4-, βB1-, and βB3-crystallin) as the abundant lens transcripts. The major difference between the guinea pig lens library, compared to the mouse and rat lens libraries, is the absence of γD-F-crystallins.

The guinea pig lens possesses a high level of ζ-crystallin (~10% of the total lens protein) containing bound NADPH [12,13]. This crystallin is a quinone oxidoreductase that was recruited to be a structural protein in the guinea pig lens [55]. ζ-crystallin is also present at high, crystallin-like levels in lenses of other species including camel (*Camelus dromedarius*) [56], rocky cavy (*Kerodon rupestris*), and degu (*Octodon degus*) [55]. It is expressed at lower levels, more appropriate for an enzymatic role, in other species, including human [57].

Two other abundant transcripts in the guinea pig lens library were lengsin and GRIFIN (galectin-related inter-fiber protein), both lens specific proteins. Lengsin was discovered as an abundant novel transcript [58] in adult human lens, while GRIFIN was discovered in the rat lens as a major lens-specific member of the galectin family [59]. The predicted protein sequence of lengsin showed significant similarity to members of the glutamine synthetase superfamily, and thus it was given the protein name of lengsin (lens glutamine synthetase-like) [60]. Lengsin is expressed in terminally differentiating fiber cells in the mouse and zebrafish lens and is implicated in the reorganization of intermediate filaments [61]. Expression of GRIFIN is limited only to differentiated fiber cells of the lens [59]. Although the function of GRIFIN is unknown, it is thought to act as a cell adhesion molecule because of its location between lens fiber cells [48,59].

The enzymes carbonic anhydrase-3 and GAPDH are fairly abundant in lenses of all species, but were particularly prominent at the cDNA level in the guinea pig lens. These enzymes belong to a group that are often abundant in lens and form a pool from which members may be recruited as structural proteins, novel crystallins, in different species [58].

Lens cytoskeletal proteins with the most abundant transcripts were phakinin (CP49, BFSP2), filensin (BFSP1), beta actin, and vimentin. Lens cytoskeletal proteins are involved in maintaining the structure and stability of lens epithelial and fiber cells, and providing elasticity during lens accommodation [62,63]. Another major transcript in the guinea pig lens was MIP/AQP0 [22]. MIP/AQP0 is the major integral membrane protein in the lens, comprising 50% of total lens membrane protein [64], and functioning as a water channel and junctional protein [65].

### 2-DE maps of guinea pig lens nuclear and cortical proteins

2-DE gels of lens nuclear and cortical soluble proteins from a 2.5-month-old guinea pig are shown in Figure 3 and Figure 4, along with identities of individual crystallins. The young lens is a tissue that contains very little water insoluble protein. In the lens nucleus, all crystallins, viz. α-crystallins (αA- and αB-crystallin), β-crystallins (βA1-, βA2-, βA3-, βA4-, βB1-, βB2-, and βB3-crystallin), γ-crystallins (γA-C-crystallin) and ζ-crystallin, with the exception of γD-F-cystallins, were detected (Figure 3). The gel contained 53 major spots, 35 of which were identified as various intact or truncated crystallins. The three missing γ-crystallins (γD-F-crystallin) either have genes deleted or missing for this developmental stage of the guinea pig lens (Figure 2).

2-DE gel analysis of the lens nucleus also indicated more αA- than αB-crystallin with a ratio of 8:1 (Figure 3). The ratio of cortical αA- to αB-crystallin was 6.5:1 (Figure 4). This guinea pig lens αA- to αB-crystallin ratio was substantially more than reported in mouse and human lenses with 2:1 and 3:1, respectively [48,66]. The cortical gel contained 47 major spots, 37 of which were identified. As expected, the older nuclear region exhibited greater amounts of truncated β-crystallins. This was especially apparent for intact βB3-crystallin, which was largely degraded in the nucleus, and replaced by a truncated acidic form above the γ-crystallin region (Figure 3).

As stated above, 2-DE gels showed more αA- than αB-crystallin. This result compares well with the relative abundance of αA-crystallin EST clones in the guinea pig lens library (466 αA- and 17 αB-crystallin). Similarly, the number of rodent lens EST clones for αA-crystallin have been reported to be more than αB-crystallin, with 60 αA- to 14 αB-crystallin for the mouse and 49 αA- to 3 αB-crystallin for the rat. In addition, the cortex contained γS-crystallin, which was either absent or expressed at very low levels in the nucleus.

### Guinea pig lens cortical crystallin masses: correlation with EST data

This EST data set permitted the assembly of many complete cDNA sequences, for prediction of protein sequence and mass. Many guinea pig lens crystallin cDNA sequences were constructed from NEIBank guinea pig ESTs (Table 2). Predicted molecular weights for the crystallins generally agreed with guinea pig intact lens cortical crystallin masses as measured by ESIMS. Although agreement between measured and calculated protein masses does not confirm sequence accuracy, disagreement frequently indicates a sequence discrepancy [67]. All crystallin mass measurements were made within an instrument mass error of 0.01% [67]. Masses were calculated after removing NH_2_-terminal methionine from all sequences, except those for αA-, αB-, αAinsert-, and βA3-crystallin, which are known to retain the methionine [47]. In addition, to account for acetylation, 42 mass units (mu) were added to the masses of each of the crystallins, with the exception of those for γA-, γB-, γC-, and γN-crystallin. Alkylation of crystallin masses (occurring as a result of treatment with iodoacetamide) was taken into account by adding 57.1 mu to each cysteine residue [47,48].

Measured masses of intact lens cortical crystallins eluted from a 2-DE gel, viz. αA-, βA2-, βA3-, βA4-, βB2-, βB3-, and γS-crystallin, matched the calculated masses based on their cDNA sequences, within a 0.01% instrument error (Table 3). For three crystallins, viz. αB-, γB-, and ζ-crystallin, the measured masses were also within 0.01% experimental error after addition of one extra oxygen atom was assumed.

**Table 3:** Guinea pig lens cortical crystallin masses (Da): calculated from cDNA sequences and measured using ESIMS.

**Crystallin**	**Calculated mass (+alkylation)**	**Notes**	**Measured mass (ESIMS)**	**Difference**	
				**mu**	**%**
alphaA	19906.1	w/ Met, w/ Acetylation	19908.9	+2.8	0.01
alphaAinsert	22575.3	w/ Met, w/ Acetylation	ND		
alphaB	20206.9	w/ Met, w/ Acetylation	20223.3	+0.4*	0.002
betaA1	23594.2	no Met, w/ Acetylation	ND		
betaA2	22433.7	no Met, w/ Acetylation	22432.7	−1.0	0.004
betaA3	25712.6	w/ Met, w/ Acetylation	25710.9	−1.7	0.007
betaA4	22609.8	no Met, w/ Acetylation	22611.8	+2.0	0.01
betaB1	27988.2	no Met, w/ Acetylation	ND		
betaB2	23418.8	no Met, w/ Acetylation	23419.0	+0.2	0.001
betaB3	24062.6	no Met, w/ Acetylation	24065.0	+2.4	0.01
gammaA	21276.7	no Met, no Acetylation	ND		
gammaB	21424.9	no Met, no Acetylation	21442.3	+1.4*	0.006
gammaC	21333.9	no Met, no Acetylation	ND		
gammaN	21577.0	no Met, no Acetylation	ND		
gammaS	21243.8	no Met, w/ Acetylation	21244.7	+0.9	0.004
zeta	35398.4	no Met, w/ Acetylation	35413.0	−1.4*	0.004

Masses of guinea pig lens cortical crystallins were determined by electrospray ionization mass spectrometry (ESIMS) after alkylation and elution of the proteins from 2-DE gels. Of a total of 29 protein spots, 15 were found to contain a sufficient amount of protein for ESIMS analysis. Ten of the 15 masses matched with calculated masses based on cDNA sequences, but 5 masses (22433.9 Da, 28065.4 Da, 22610.8 Da, 23802.9 Da, and 22388.7 Da) could not be matched. Six crystallins, which were known to be present in the guinea pig lens based on the cDNA sequences (Table 3), could not be matched with measured masses determined by ESIMS (labeled as ND, not determined). All measured masses were within an instrument error of 0.01%. Theoretical masses were calculated after removing the NH_2_-terminal methionine from all sequences (except those for alphaA, alphaAinsert, alphaB and betaA3). A mass unit of 42 was added to the masses of all crystallins (except those for gammaA, gammaB and gammaN) to account for the N-terminal acetyl group. Alkylated protein mass was accounted for by adding 57.1 mass units to each cysteine residue. The asterisk indicates that 16 mu was subtracted based on the assumption of the addition of one oxygen atom.

Six crystallins (αAinsert-, βA1-, βB1-, γA-, γC-, and γN-crystallin, which are labeled ND in Table 3) were not detected and their calculated masses did not match any of the measured masses of proteins isolated and analyzed by ESIMS. This may have been due to an insufficient amount of protein or poor recovery during the analysis of these six crystallins. Finally, of the 15 protein spots analyzed, five masses measured by ESIMS, viz. 22433.9 Da, 28065.4 Da, 22610.8 Da, 23802.9 Da, and 22388.7 Da, could not be matched with any of the crystallins, based on cDNA sequences. It is possible that the unmatched proteins are crystallins or other lens proteins with post-translational modifications.

### Guinea pig retina cDNA library (naz)

**Figure 6:**
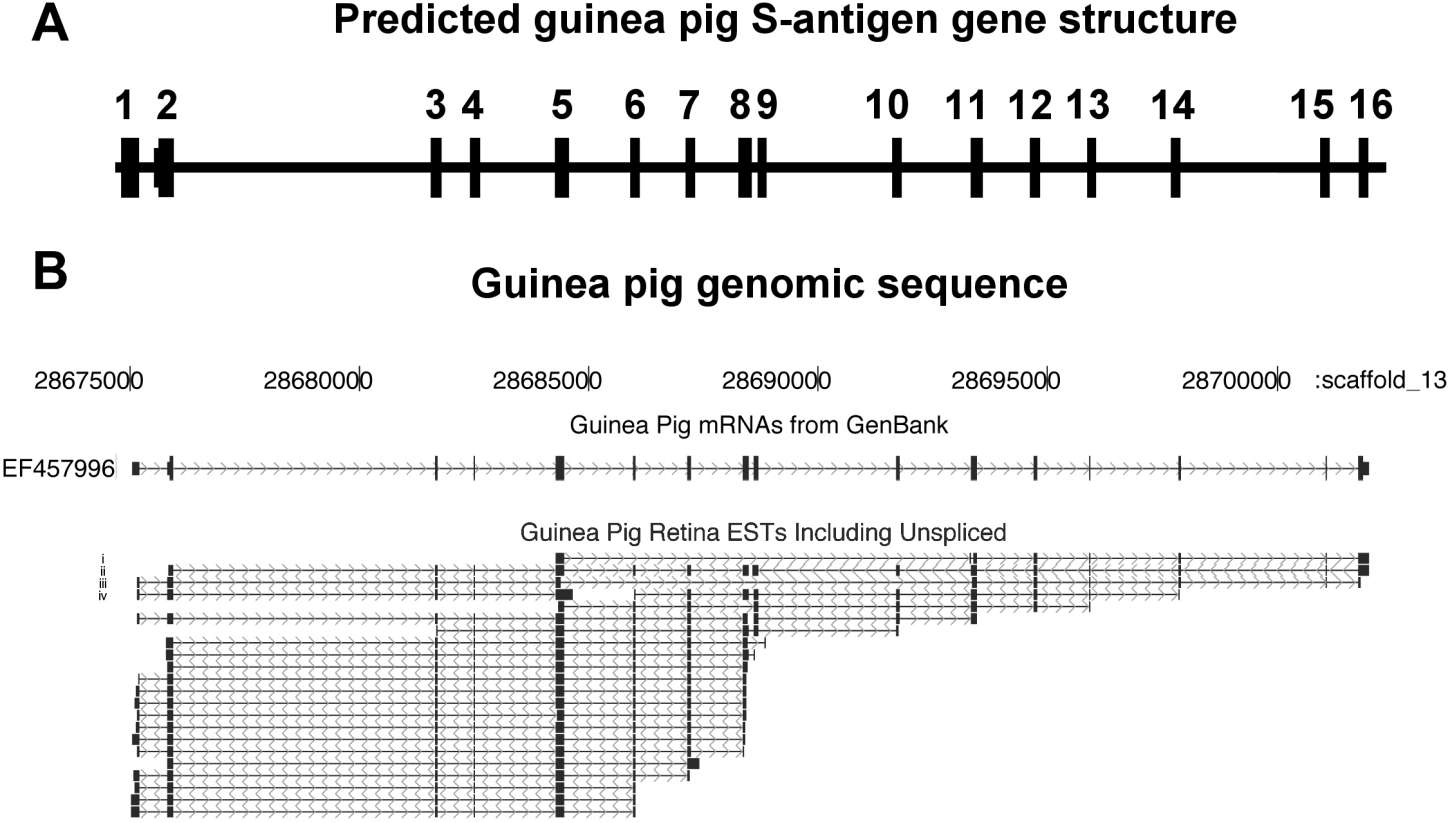
Alternate splicing in the guinea pig *S-antigen* gene. **A**: Predicted guinea pig *S-antigen* gene structure from guinea pig retina *S-antigen* ESTs. **B**: ESTs aligned to guinea pig genome (scaffold_13) and guinea pig mRNA from GenBank for *S-antigen* (*Sag, Arrestin*) (EF457996) generated from NEIBank ESTs. *Sag* shows that some exons are absent, for example: EST (i) and (iii) are missing exons 6, 7, 8, 9, and 10, EST (ii) and (iv) may terminate early at exons 8 and 5. Note: arrows show introns in the direction of sequence reads.

The percentage of gene clusters of the guinea pig retina cDNA library having significant homology to mammalian (non-guinea pig) GenBank sequences was 56%. In comparison, human and mouse retina cDNA libraries had 80% [40] and 85% [54] significant GenBank homology, respectively. While the majority of ESTs correspond to canonical gene transcripts, some genes show evidence of relatively frequent alternative (or aberrant) splicing. As an example from retina, *S-antigen* (*Arrestin*) spliced ESTs and guinea pig mRNAs are shown aligned to the guinea pig genome scaffold_13 (Figure 6). A surprising number of alternatively spliced transcripts are evident, in particular different patterns of exclusion of exons 6–10. For example, EST (i) and (iii) are missing exons 6, 7, 8, 9, and 10 while EST (ii) and (iv) may terminate early at exons 8 and 5, respectively. Whether this has functional significance remains to be seen, but since several of the variants interrupt the open reading frame and have stop codons ahead of the last exon, they would be subject to nonsense-mediated decay and would not produce proteins. This might have a regulatory role for levels of S-antigen or may simply reflect inefficient splicing of an abundant mRNA. Such exon skipping is also apparent in human *S-antigen* ESTs, but at a lower frequency.

Photoreceptor transcripts were among the most abundant in the guinea pig retina library (Table 4), as were genes encoding proteins essential for retina development, such as Rhodopsin, S-antigen (Sag, arrestin), beta-transducin-1 (Gnb1), alpha-transducin-1 (Gnat1), NRL (neural retina leucine zipper), phosducin, peripherin-2, phosphodiesterase-6-gamma subunit (Pde6g), guanylate cyclase activator-1b (Guca1b), and retinitis pigmentosa RP1 protein homolog (oxygen-regulated protein 1). Photoreceptor-specific transcripts are the most abundant transcripts in mammalian retinal cDNA libraries, including the NEIBank mouse and rat retinal EST libraries [54]. Other fairly abundant transcripts in both the guinea pig and mouse retina libraries were enolase, aldolase, Gapdh, and elongation factor alpha [54]. The rat and guinea pig retina libraries had some similar photoreceptor transcripts, such as Rhodopsin, α-transducin, S-antigen, and NRL. Other retinal transcripts abundant in the rat EST library but absent in the guinea pig EST library were rod outer segment membrane protein 1, ferritin heavy polypeptide 1, glycoprotein, synaptic 2, Ybx protein 1, and solute carrier family 17.

**Table 4:** Most abundant guinea pig retina cDNA transcripts (naz).

**Rank**	**GenBank description**	**N**
1	rhodopsin (Rho)	64
2	S-antigen (Sag, Arrestin)	24
3	beta-transducin (Gnb1)	22
4	aldolase C (Aldoc)	21
5	glyceraldehyde 3-phosphate dehydrogenase (Gapdh)	14
6	alpha-transducin (Gnat1)	12
7	violet-sensitive visual pigment (SWS1)	9
8	elongation factor 1 alpha	9
9	glutamine synthetase (Glul)	9
10	neural retina leucine zipper transcription factor (NRL)	8
11	phosducin (PHD)	8
12	creatine kinase B (B-CK)	8
13	alpha-enolase (Eno1)	7
14	unc-119 (Unc119)	7
15	aryl hydrocarbon receptor interacting protein-like 1 (Aipl1)	6
16	carboxypeptidase E (Cpe)	6
17	probable 3′ UTR of Gnb1	6
18	peripherin-2 (rds) (Prph2)	6
19	synaptosomal-associated protein 25 (Snap25)	5
20	ubiquitin C (Ubc)	5
21	phosphodiesterase 6 gamma subunit (Pde6g)	5
22	alpha transducin (cone) (Gnat2)	5
23	glucose-6-phosphatase 2 (G6pc2)	5
24	guanylate cyclase activator 1B (Guca1b)	5
25	ATP synthase, H^+^ transporting F1alpha (atp5a1)	5
26	heat shock 70 kDa protein 8 (Hspa8)	5
27	pyruvate kinase 3 (Pkm2)	5
28	actin gamma1 (Actg1)	
29	eukaryotic translation initiation factor 4A2 (Eif4a2)	4
30	testis enhanced gene transcript (Bax inhibitor 1) (Tegt)	4
31	histone H3.3A (H3f3a)	4
32	tubby like protein 1 (Tulp1)	4
33	guanylate cyclase activator 1a (Guca1a)	4
34	small nuclear ribonucleoprotein polypeptides B and B1 (Snrpb)	4
35	N-myc downstream regulated gene 1 (Ndrg1)	4
36	transferrin (Tf)	4
37	retinitis pigmentosa 1 (RP1)	4
38	1-acylglycerol-3-phosphate O-acyltransferase 3 (Agpat3)	4

Genes corresponding to the most abundant transcripts (≥4 clones) in 2.5-month-old guinea pig (*Cavia porcellus*) retina are listed and ranked by abundance. The total number of sequenced clones in each gene cluster (N) is listed. GenBank descriptions are based on clusters having significant homologies to mammalian Genbank sequences.

Several retina transcripts are orthologs of human retinal disease genes [68,69]. About 30% of autosomal dominant retinitis pigmentosa (AD_RP) is caused by mutation of the *Rhodopsin* gene [70]. *Nrl*, a rod-photoreceptor specific member of the maf family of bZIP-domain transcription factors, was quite abundant in the guinea pig retina library (Table 4). *NRL* transcripts are also abundant in the human and rat retina [71] and mutations to *NRL* cause AD-RP [68]. Missense mutations in the gene encoding alpha-transducin-1 (GNAT1) protein are known to produce autosomal dominant congenital stationary night blindness [72]. Several other retina transcripts, particularly those involved in phototransduction, are associated with inherited retinal diseases in humans [68,69]. The PDE6G enzyme cleaves cGMP required for the opening of cation channels in rod photoreceptors [67] and mutations affecting *PDE6G* also result in retinal degenerations [71].

The guinea pig retina library also contained clones for the violet sensitive visual pigment (Sws1). This gene encodes a visual pigment with absorbing wavelengths of 390–450 nm for violet. In general, the transcriptome of guinea pig retina is similar to that of human [71]. Transcripts absent from the guinea pig retina library, which were reported as abundant in human retina, include Glutathione Peroxidase (GSHPx) and Prostaglandin D Synthetase [71]. The human retina, in contrast to the guinea pig, has an extensive retinal vasculature [27], and it is possible the above two enzyme transcripts are derived from blood cells.

### Guinea pig eye minus lens and retina cDNA library (nba)

**Figure 7:**
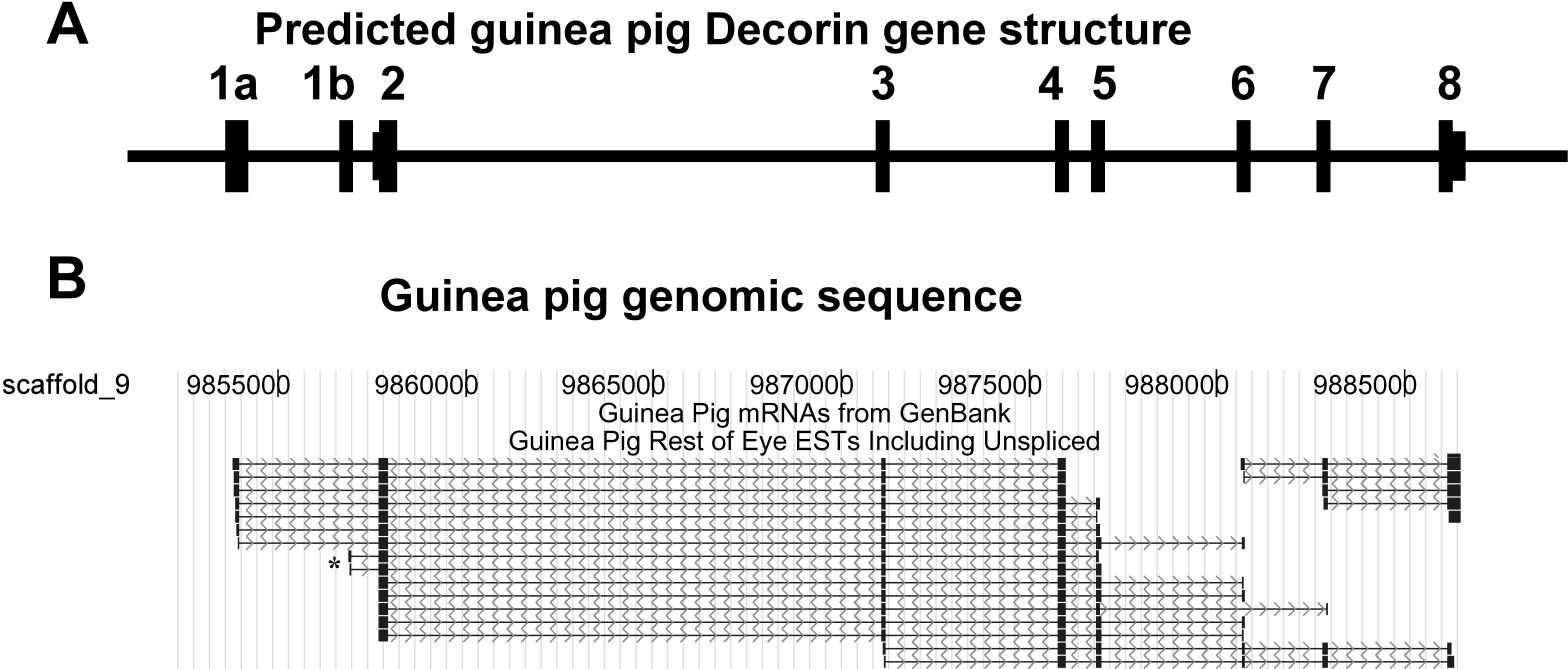
Alternate splicing of the guinea pig *Decorin* gene. **A**: Predicted guinea pig *Decorin* (*Dcn*) gene structure from guinea pig “rest of the eye” Decorin ESTs. **B**: ESTs aligned to guinea pig genome (scaffold_9) and guinea pig mRNA from GenBank for *Dcn* generated from NEIBank ESTs. *Dcn* gene shows a minor alternative first exon (alternative transcription start site; a black asterisk shows two cDNAs which contain an alternative exon with a potential protein coding open reading frame) that is also seen in other species [45]. Note: arrows show introns in the direction of sequence reads.

Eye tissue for the guinea pig eye minus lens and retina library (nba) consisted of the cornea, iris, ciliary body, trabecular meshwork, choroid, sclera, and RPE (Figure 1B-D). This library contained some retinal content, including clones for Rhodopsin (8 clones in this library versus 64 in the retina library), and S-antigen (3 clones versus 24 in retina). This results from the difficulty in avoiding some neural retina contamination in the dissection. Most of the abundantly expressed genes identified were logical markers for cornea, RPE/choroid, and sclera. Several genes for extracellular matrix and glycoproteins, which are abundant in cornea, sclera, and choroid, were also observed (Table 5). Examples include decorin, annexin A1 (Lipocortin-like protein 39 kDa), Collagen alpha-2 type I, Sparc and Keratin 12. For Decorin (*Dcn*), EST evidence indicated an alternative 5′-exon and as a possible alternative transcription start site (Figure 7B, shown with a black asterisk) which is also apparent in some human mRNAs [73,74]. Decorin ESTs and mRNA were aligned with scaffold_9 of the guinea pig genome (Figure 7). No guinea pig reference mRNAs were available during production of this alignment.

**Table 5:** Most abundant guinea pig eye minus lens and retinal transcripts (nba).

**Rank**	**GenBank description**	**N**
1	decorin (DCN)	21
2	elongation factor 1 alpha (Eef1a1)	14
3	keratin 12 (Krt12)	11
4	annexin A1 (lipocortin-like protein 39K) (anaxa1)	10
5	beta-transducin (Gnb1)	9
6	rhodopsin (Rho)	8
7	collagen alpha-2(I) (Col1A2)	8
8	ribosomal protein S3a	6
9	secreted protein, acidic, cysteine-rich (Sparc)	6
10	glyceraldehyde-3-phosphate dehydrogenase (Gapdh)	5
11	Y box binding protein 1 (Ybx1)	5
12	apolipoprotein D (Apod)	5
13	prosaposin (Psap)	5
14	ribosomal protein L7 (RPL7)	5
15	apolipoprotein E (Apoe)	5
16	ribosomal protein S3 (Rps3)	5
17	retinal pigment epithelium-specific protein 65 kDa (Rpe65)	5
18	phosducin (PDC)	4
19	aldolase C (Aldoc)	4
20	ribosomal protein L14 (Rpl14)	4
21	creatine kinase B (B-CK)	4
22	adipocyte enhancer binding protein 1 (Aebp1)	4
23	CD9 (Tspan29) (CD9)	4
24	aldehyde dehydrogenase 3A1 (Aldh3a1)	4
25	thymosin beta 4, X-linked (Tmsb4x)	4

Genes corresponding to the most abundant transcripts (≥4) in the eye minus lens and retina library (nba) of a 2.5-month-old guinea pig (*Cavia porcellus*) are listed and ranked by abundance. The total number of sequenced clones in each gene cluster (N) is indicated. GenBank descriptions are based on clusters having significant homologies to mammalian Genbank sequences.

The RPE-specific protein RPE-65 was also abundant, as was apolipoprotein E (APOE) precursor. RPE65 is an enzyme that converts *trans* retinyl esters into 11-*cis* retinal, which is essential for the visual cycle to recycle *cis*-retinal back to the photoreceptors [75]. Mutations or loss of *RPE-65* are also associated with retinal degenerations in mice and humans [76]. Polymorphisms of *APOE* are presently of great interest for their association with human age-related macular degeneration [76]. APOE is a major apolipoprotein and regulates lipid and cholesterol transport in the central nervous system [77].

Abundant markers for the cornea were keratin 12, decorin and aldehyde dehydrogenase class 3 (Aldh class 3) and collagen alpha-2 type I, a major structural component of the cornea [69]. The same proteins have also been reported to be abundant in the human cornea cDNA library [73]. Other abundant transcripts included a transcriptional regulator Y-box binding protein 1 (Ybx1), ribosomal protein S3a, and glyceraldehyde-3-phosphate dehydrogenase (Gapdh).

The mouse cornea and RPE/choroid libraries, unlike the rat iridocorneal library, shared most transcripts with guinea pig eye minus lens and retina (“rest of the eye”) library (Table 5), such as decorin, elongation factor 1 alpha, keratin 12, Rhodopsin, Sparc, prosaposin, Apoe, aldehyde dehydrogenase 3A1, and ribosomal proteins. In the rat iridocorneal library, the most abundant transcripts but absent in the guinea pig library were β-actin, ribosomal protein S2 isoform 7, insulin-like growth factor, basigin, dopachrome tautomerase, orthine decarboxylase antizyme 1, eukaryotic elongation factor 1 α-1, ferritin heavy, and keratin 2, with the exception of Apoe present in both rat and guinea pig libraries.

This guinea pig eye minus lens and retina library and the NEIBank human trabecular meshwork library [78] share 16 transcripts that are abundant in both libraries, with the most abundant being ribosomal protein S3a with 6 cDNAs (Table 5). Other shared transcripts were Decorin, Keratin 12, Collagen alpha-2 type I, Sparc (Osteonectin), Y box binding protein 1, Apolipoprotein D, Aldehyde Dehydrogenase class 3, thymosin beta-4, and Prostaglandin-D2 (PGD2) Synthase (<4 clones, therefore not included in Table 5, but present in NEIBank eye minus lens and retina cDNA library). PGD2 synthase is also abundant in the cDNA library for human iris [79]. This enzyme, PGD2 synthase, is responsible for the synthesis of prostaglandin D, which has been implicated in the control of intraocular pressure [80].

In conclusion, the guinea pig is an important model organism in several areas of modern eye research. What was lacking is a characterization of the transcriptional repertoire of guinea pig eye tissues and a definition of the full sequences of key proteins from lens, retina and other parts of the eye. Here we describe three new cDNA (EST) libraries for tissues of the guinea pig eye. These provide sequence verified cDNA clones for future studies and complete sequence information for many eye proteins. The guinea pig has its own pattern of similarities and differences with the human eye and provides an important alternative to other research models. EST analyses have already illustrated the differences between the transcriptomes of human and murine rodent eye tissues [71]. Many eye genes appear to have similar structures and splicing variants as their human and mouse counterparts. In particular we have characterized the complete set of guinea pig crystallins, and have verified most of these by mass spectrometry. Surprisingly, our results suggest that the γD-F-crystallin genes, which are found in other mammalian genomes, may actually be deleted or the expression of these genes missing from the guinea pig genome for this developmental stage of the guinea pig lens. Although γD-crystallin is abundant in many species, no clones for γD-, γE-, or γF-crystallin were found, and no equivalent genomic sequences were found in the current guinea pig genome. This data set is also an important contribution of novel ESTs, which are needed to support gene structural annotation of the first draft of the guinea pig genome.
